# Nanoporous Gold Nanocomposites as a Versatile Platform for Plasmonic Engineering and Sensing

**DOI:** 10.3390/s17071519

**Published:** 2017-06-28

**Authors:** Fusheng Zhao, Jianbo Zeng, Wei-Chuan Shih

**Affiliations:** 1 Department of Electrical and Computer Engineering, University of Houston, 4800 Calhoun Rd, Houston, TX 77004, USA; fzhao3@uh.edu (F.Z.); jzeng8@uh.edu (J.Z.); 2 Department of Biomedical Engineering, University of Houston, 4800 Calhoun Rd, Houston, TX 77004, USA; 3 Program of Materials Science and Engineering, University of Houston, 4800 Calhoun Rd, Houston, TX77004, USA; 4 Department of Chemistry, University of Houston, 4800 Calhoun Rd, Houston, TX 77004, USA; 5 Biomedical Institute for Global Health Research and Technology (BIGHEART), National University of Singapore 14 Medical Drive, Singapore 117599, Singapore

**Keywords:** nanoporous gold disk, nanoporous nanocomposite, surface-enhanced Raman scattering, plasmonic sensing

## Abstract

Plasmonic metal nanostructures have shown great potential in sensing applications. Among various materials and structures, monolithic nanoporous gold disks (NPGD) have several unique features such as three-dimensional (3D) porous network, large surface area, tunable plasmonic resonance, high-density hot-spots, and excellent architectural integrity and environmental stability. They exhibit a great potential in surface-enhanced spectroscopy, photothermal conversion, and plasmonic sensing. In this work, interactions between smaller colloidal gold nanoparticles (AuNP) and individual NPGDs are studied. Specifically, colloidal gold nanoparticles with different sizes are loaded onto NPGD substrates to form NPG hybrid nanocomposites with tunable plasmonic resonance peaks in the near-infrared spectral range. Newly formed plasmonic hot-spots due to the coupling between individual nanoparticles and NPG disk have been identified in the nanocomposites, which have been experimentally studied using extinction and surface-enhanced Raman scattering. Numerical modeling and simulations have been employed to further unravel various coupling scenarios between AuNP and NPGDs.

## 1. Introduction

In recent years, the intriguing optical properties of metallic nanostructures have become a research focus. Light-excited collective oscillation of conduction-band electrons in metallic nanostructures is known as surface plasmon polariton (SPP) for propagating ones and localized surface plasmon resonance (LSPR) for non-propagating ones. Both SPP and LSPR exhibit a significant dependence on the near-field environment in the close proximity to the nanostructures. Local changes in the dielectric function can significantly modulate the resonance frequency, which has become an effective means for molecular sensing [1,2,3]. For example, the proximity influence of target molecules can be considered as a slight increase in the local refractive index against the original index in either the air or water. When the proximity entity is another metal nanostructure or nanoparticle, plasmonic coupling can occur and causes pronounced modulation in the combined optical properties. Plasmonic coupling between nanostructures can lead to resonance frequency shifts, plasmonic hybridization, “hot-spots” generation, changes in radiation damping, etc. [4,5,6,7,8]. From the design and optimization point of view, plasmonic coupling can provide an alternative means for plasmonic engineering and sensing. Plasmon-induced electric field (E-field) localization has been recognized as the primary mechanism in surface-enhanced spectroscopy, such as surface-enhanced Raman scattering (SERS) [9,10], surface-enhanced fluorescence (SEF) [11], surface-enhanced infrared absorption (SEIRA) [12,13], and surface-enhanced near-infrared absorption (SENIRA) [14].

The performance of surface plasmon-based techniques depends on a well-designed plasmonic substrate with desirable plasmonic properties. For instance, photothermal therapy requires the resonance peak position to be inside the near-infrared region, also known as the “diagnostic/therapeutic window”, for deeper tissue penetration [15]. The performance of surface-enhanced spectroscopy is typically better when the LSPR resonance aligns with the excitation and/or the emission/scattering wavelengths [16]. In addition to placing the LSPR resonance at the desired wavelength, plasmonic coupling-induced “hot-spots” provide additional E-field enhancement to further intensify light-matter interactions [17,18,19,20]. The coupling between closely located nanoparticles was shown to be efficient for both purposes: the near-field coupling can effectively shift the LSPR peak position [21] and the nano-gaps between particles can generate strong E-field due to gap-mode resonance [22,23].

Lithographically patterned sub-micron nanoporous gold disk (NPGD) features large surface area and high-density hot-spots [24]. It has been demonstrated in DNA cancer marker detection by both SERS and SEF [25,26], label-free sensing and imaging of physiological small analytes for disease diagnosis [27], photothermal inactivation of pathogens [28,29], and chemical analysis by SENIRA [14]. In addition to providing highly enhanced spectroscopy sensing capabilities, NPGDs also have plasmonic properties that can be tuned by varying their external geometrical features via lithographic patterning and internal nanoporous morphology via controlled dealloying [30], laser and furnace annealing [30,31], and surface modifications [32].

In this work, we demonstrate a versatile platform for plasmonic engineering and sensing by loading colloidal nanoparticles onto NPGD. Gold nanoparticles (AuNP) with different sizes are loaded onto NPGD substrates to form hybrid nanocomposites, which exhibit red-shifted resonance peaks compared to bare NPGD. From the material design aspect, the magnitude of the coupling-induced shift is larger than other approaches described previously [30,31,32]. The plasmonic resonance is tunable in the near infrared (NIR) spectral region by changing the AuNP size. Finite-difference time-domain (FDTD) simulation reveals that coupling-induced hot-spots not only originate from the interaction between NPGD and AuNP, but also from between AuNPs. From the sensing aspect, the platform can be employed to detect AuNP when they are in the proximity. In addition, potential applications of nanocomposites in molecular sensing by SERS will be illustrated using 3,3′-diethylthiatricarbocyanine iodide (DTTC) molecule. The results suggest that the nanocomposites exhibit improved SERS sensitivity and can be used as novel sensors and imaging labels [33].

## 2. Materials and Methods

### 2.1. Materials

Chloroform (anhydrous, ≥99.0%), nitric acid (ACS reagent, 70%), poly(diallyldimethylammonium chloride) (PDDA, 20 wt. % in H_2_O), 3,3′-diethylthiatricarbocyanine iodide (DTTC, 99%), sodium citrate dehydrate (≥99.0%), gold (III) chloride hydrate (99.999% trace metals basis), sodium dodecyl sulfate (ACS reagent, ≥99.0%) and latex beads (polystyrene beads (PS beads), 10% aqueous suspension) with mean particle sizes 460 nm were purchased from Sigma-Aldrich. Ethanol (200 proof) was from Decon Laboratories, Inc. Silicon wafers were obtained from University Wafers, and coverglass (22 mm × 40 mm, No. 1) from VWR. Ag_70_Au_30_ (atomic percentage) alloy sputtering targets were purchased from ACI Alloys, Inc. Argon gas (99.999%) was used for RF-sputter etching; 50 nm AuNP were purchased from BBI Solutions.

### 2.2. Characterization

Scanning electron microscope (SEM) images were obtained from PHILIPS FEI XL-30 FEG-SEM system. A Cary 50 Scan UV-visible spectrometer and a Jasco V-570 UV-Vis-NIR spectrophotometer were used to measure extinction spectra of the monolayer NPGDs and nanocomposites on a glass coverslip. The SERS spectra of DTTC were recorded by using a home-built line-scan Raman microscope, and the automated image curvature correction algorithm was employed, followed by 5th-order polynomial background removal [34].

### 2.3. Fabrication of Nanoporous Composites

**Figure 1:**
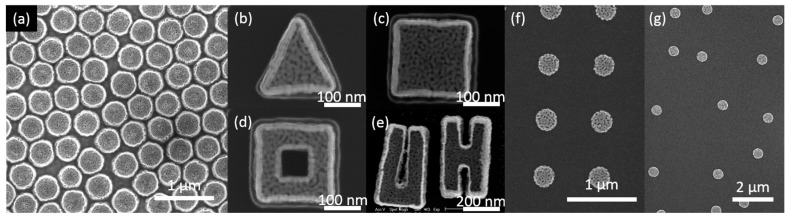
SEMs of nanoporous gold (NPG) nanoparticles fabricated by various methods. (**a**) nanoporous gold disk (NPGD) array fabricated by a method based on nanosphere lithography; (**b**–**e**) Arbitrary NPG nanoparticles; (**f**) Periodic NPGD array; and (**g**) Random NPGD array fabricated by a method based on electron-bean lithography (EBL).

Several fabrication methods for NPG nanoparticles have been reported in the literature; these methods are based on dewetting process, [35,36] nanosphere lithography technique, [24] and electron-bean lithography (EBL) technique [37]. The dewetting process-based fabrication method produces NPG nanoparticles highly irregular in shape and size, whereas the latter two produce well-controlled nanoparticles. With nanosphere lithography-based fabrication process, a monolayer of polystyrene micron-beads is used as etching template for the patterning of silver–gold alloy nanodisks which are further processed into NPG nanoparticle through dealloying process [24]. Such a method produces monodispersed monolithic NPG nanodisk high-density arrays as shown in Figure 1a. The EBL-based method provides even better control over the nanoparticle shape, size and locations. With such a technique, the nanoparticles are fabricated by EBL and lift-off process. The silver–gold alloy composition for the precursor nanoparticle is achieved by the evaporation of alternating silver and gold thin layers followed by thermal annealing. This method is capable of fabricating arbitrary-shaped NPG nanoparticle (Figure 1b–e) with a predetermined location such as periodic arrays or random arrays (Figure 1f,g).

**Figure 2:**
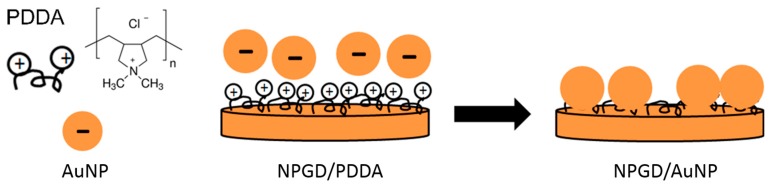
The strategy to synthesize NPG nanocomposites.

In this study, the NPGD are fabricated through nanosphere lithography-based method. Fabrication conditions produce as-prepared NPGDs with ~300 nm diameter, ~75 nm thickness and ~8.5 nm pore size on average. The NPG nanocomposites were synthesized by taking advantage of the electrostatic interaction mechanism that occurs between negatively charged AuNPs and positively charged NPGD surfaces. Such a method provides an easy route for the attachment of AuNPs compared with covalent attachment, since the molecules used for positively charging the NPGD substrate are readily available. As shown in Figure 2, NPGDs were functionalized with PDDA to achieve positively charged surfaces. Negatively charged AuNPs with ~13 nm and ~50 nm mean diameter with <10% coefficient of variation were prepared by Turkevich protocol [38,39], or purchased from BBI Solutions, respectively. AuNPs were washed one time by centrifugation to remove negatively charged surfactants and concentrated 10 times. Positively charged NPGDs were then incubated in negatively charged AuNPs colloidal solutions for ~15 h to form nanocomposites. After incubation, the samples were thoroughly washed with deionized (DI) water and subsequently dried by nitrogen flow.

## 3. Results and Discussion

### 3.1. Characterization of Nanoporous Composites

**Figure 3:**
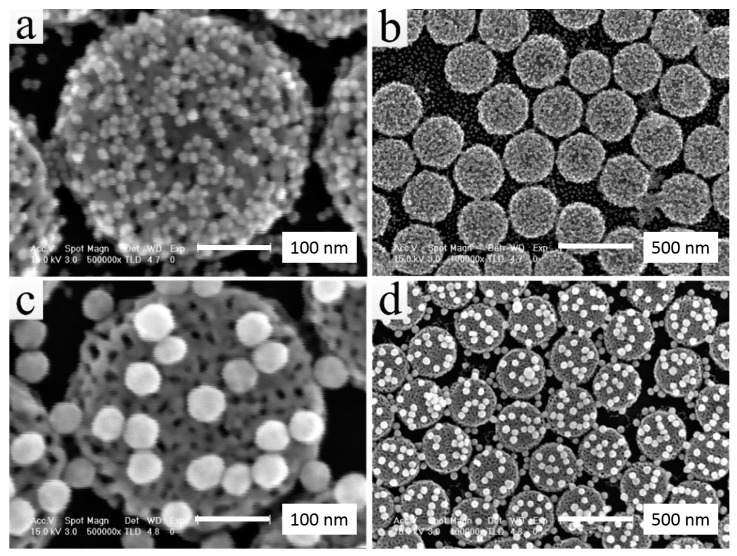
SEM images of NPGD/AuNP composites: (**a**) and (**b**) ~13 nm AuNPs on NPGD; (**c**) and (**d**) ~50 nm AuNPs on NPGDs. AuNP: gold nanoparticles.

SEM images of NPGD/AuNP composites are shown in Figure 3 where 13 nm AuNPs were employed in (a) and (b) and 50 nm AuNPs in (c) and (d). For a fair comparison, it is desired that AuNPs of both sizes are attached on the surfaces of NPGDs instead of loaded into the pores. Therefore, the alloy (Ag70at.%Au30at.%) disks were dealloyed in 68% nitric acid for 1 min to obtain an average pore size of ~8.5 nm. As shown in Figure 3a, 13 nm AuNPs attached to NPGDs with a higher density and formed structures such as dimers, trimers, tetramers and assembled clusters. The particle density of attached 50 nm AuNPs on NPGDs (Figure 3c) is much lower than that of the 13 nm (Figure 3a). At a low magnification (Figure 3b,d), both SEM images show the inherent spatial heterogeneity of AuNPs on NPGDs due to the random attaching process.

**Figure 4:**
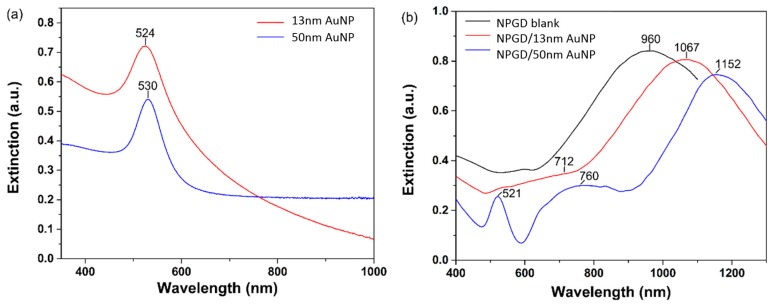
Extinction spectra: (**a**) AuNPs; and (**b**) NPGD/AuNP composites.

The plasmonic properties of NPGD/AuNP composites were investigated in comparison with AuNPs and NPGDs (Figure 4). As shown in Figure 4a, 13 nm and 50 nm AuNPs in water exhibit extinction peaks at 524 and 530 nm, respectively [40]. NPGD with ~300 nm diameter has the extinction peak at 960 nm in air (Figure 4b), which corresponds to the in-plane dipole resonance mode. The peak position red-shifted to 1067 nm when the NPGDs are attached with 13 nm AuNPs, and red-shifted to 1152 nm upon attaching 50 nm AuNPs. It is noted that the plasmon resonance of NPGD is sensitive to the refractive index changes [24]. Therefore, the peak shift caused by adsorbed PDDA was investigated. As shown in Figure S1, the extinction peak of NPGD red-shifted ~15 nm after it was functionalized with PDDA. Apparently, the much stronger red-shifts induced by the adsorption of AuNPs originated from the interaction between NPGDs and AuNPs. In our previous work [24], we reported that the strong plasmonic coupling generated between the nanoporous structures and the patterned disk shape significantly red-shifts the in-plane dipole resonance peak. On the other hand, a decrease of the plasmonic coupling caused by the increased pore and ligament size induces blue-shift [30]. Qian et al. demonstrated a giant SERS enhancement primarily due to strong coupling between nanopore and AuNPs, which was further confirmed by FDTD simulation [41]. In this work, the red-shifts of NPGD/AuNP composite extinction peaks also indicate a strong plasmonic coupling between AuNPs and NPGDs. The interaction between NPGDs and 13 nm AuNPs induced ~92 nm red-shift of the extinction peak, whereas 50 nm AuNPs induced ~177 nm red-shift. To our knowledge, this is the first study to demonstrate the effect of AuNP size on its coupling strength with plasmonic nanoporous materials.

**Figure 5:**
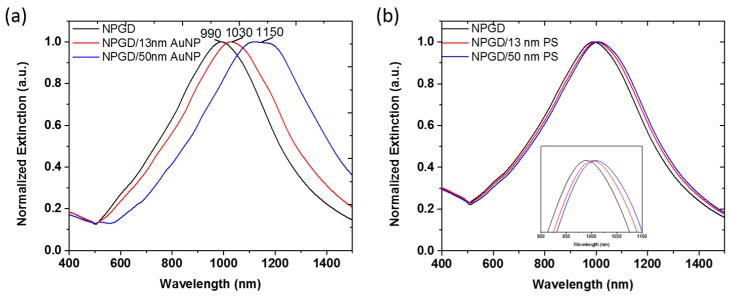
FDTD-simulated extinction spectra. (**a**) 300 nm blank NPGD, NPGD/13 nm AuNP composite and NPGD/50 nm AuNP composite; (**b**) Same setups as (a) with AuNP replaced by polystyrene (PS) beads of the same size. FDTD: finite-difference time-domain.

To further elucidate the red-shift behavior of AuNP on NPGD, we performed FDTD simulations. The 3D NPGD structure used in the simulation is generated using 2D SEM images. As shown in Figure 5a, the simulated LSPR peak for NPGD is at 990 nm, NPGD/13 nm AuNP composite at 1030 nm, and NPGD/50 nm AuNP composite at 1150 nm. The trend of the peak red-shift is consistent with experimental results. The extinction spectra of NPGD/AuNP composites suggest that the loading of AuNPs could markedly influence the LSPR of NPGD in the NIR region. To show such red-shift is indeed caused by coupling between NPGD and AuNP, similar simulations were carried out with AuNPs replaced by PS beads. As shown in Figure 5b, the PS beads-induced refractive index increase had limited effect on the peak position of the LSPR of NPGD.

**Figure 6:**
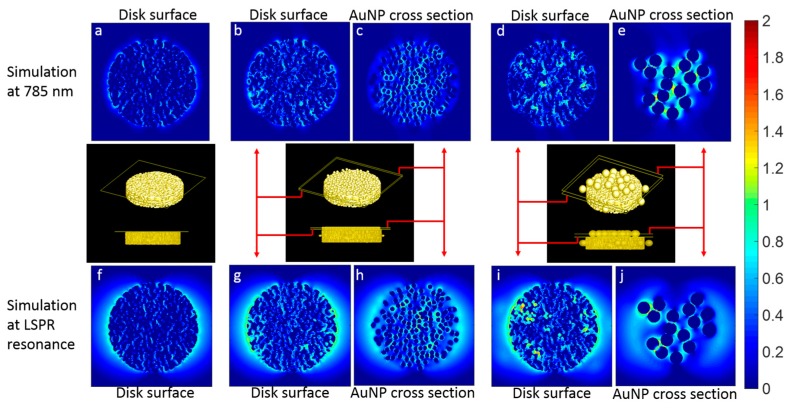
FDTD simulation results of E-field distribution: (**a**,**f**) blank NPGD; (**b**,**c**,**g**,**h**) NPGD/13 nm AuNP composite; and (**d**,**e**,**i**,**j**) NPGD/50 nm AuNP composite. The E-field distributions are calculated for both the excitation laser wavelength (785 nm, a–e) used in SERS measurements and the LSPR wavelength for various nanocomposites: NPGD (990 nm, f), NPGD/13 nm AuNP (1030 nm, g,h) and NPGD/50 nm AuNP (1150 nm, i,j). The scale bar is the log10 of the relative E-field intensity.

The E-field distributions of the nanocomposites were also simulated with FDTD as shown in Figure 6. In comparing the simulation results at 785 nm (Figure 6a–e) with the ones obtained at the resonance wavelength (Figure 6f–j), the E-field is stronger for the latter ones, as expected. Also, it is clear that the hot-spots are more concentrated at the disk center with 785 nm wavelength, whereas in the case of resonance wavelength, the hot-spots have moved to the left and right edges of the disk. This is simply due to the fact that the dominating LSPR resonance mode is the dipolar disk mode resonance, and thus the mode shape.

Furthermore, by comparing the E-field distribution of the NPGD and NPGD/AuNP composites, it is readily seen that additional hot-spots exist in the composites. The additional hot-spots at the NPGD surface indicate a strong coupling between NPGD and AuNP, whereas the hot-spots between AuNPs indicate a strong coupling among AuNPs.

Finally, by comparing the E-field distribution of the NPGD/13 nm AuNP composites with the NPGD/50 nm AuNP composites, it is observed that the 13 nm AuNP generated a higher number of hot-spots due to the higher particle density, whereas the 50 nm AuNP has a lower number of hot-spots but stronger intensity due to a stronger coupling. The stronger coupling for 50 nm AuNP corresponds well with the stronger red-shift in the extinction spectra.

**Figure 7:**
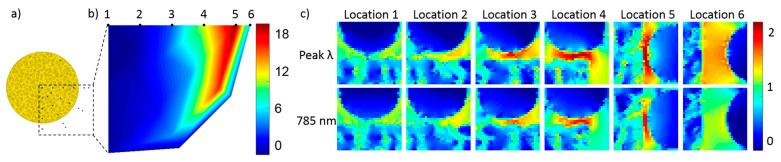
Simulation result for coupling between a single NPGD and a single AuNP. The locations of the single AuNP with respect to the NPGD are indicated in (**a**); the peak shift induced by the AuNP at different locations are plotted as a colormap in (**b**); and (**c**) the E-field distribution between the AuNP and the NPGD at location 1 to 6 indicated in (b); The scale bar is the log10 of the relative E-field intensity.

Another set of simulations were conducted for a single 50 nm AuNP located on or near a NPGD at different positions. Such simulations reveal the fact that the coupling strength and the amount of peak shift induced by nanoparticles are highly location dependent. The simulated center positions of the 50 nm AuNP are indicated in Figure 7a. When the center is within the NPGD boundary, the AuNP is located above the NPGD with 2 nm separation, otherwise, the AuNP is located parallel to the NPGD. The amount of peak shifts induced by the single AuNP at different locations are analyzed and plotted into a colormap as shown in Figure 7b. The colormap corresponds well with the E-field distribution of the NPGD at its resonance wavelength. That is, when the AuNP is located inside the enhanced E-field generated by NPGD, strong coupling effect can occur. The hot-spots induced by single AuNP at different locations are also being investigated. The E-field at six locations along the polarization direction ranging from the center to out of the NPGD boundary are plotted in Figure 7c. These figures clearly show a higher E-field enhancement when the AuNP is located near the edge of the NPGD, which corresponds well with the stronger coupling observed earlier. The results shown here clearly indicated that the AuNPs located close to the edges contribute more to the peak shift and hot-spots generation. Notice that, here the hot-spots are generated between AuNP and NPGD; the hot-spots generated between AuNPs should not be affected by the particle position.

### 3.2. Probing Hot-Spots by Surface Adsorbates

**Figure 8:**
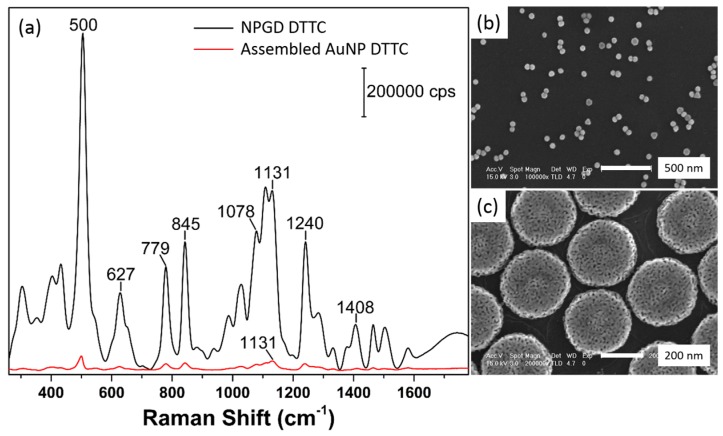
(**a**) SERS spectra of DTTC on NPGD and 50 nm AuNPs; (**b**) SEM image of 50 nm AuNPs assembled on Si wafer, scale bar 500 nm; (**c**) SEM image of 300 nm NPGD on Si wafer, scale bar 200 nm. SERS: surface-enhanced Raman scattering; DTTC: 3,3′-diethylthiatricarbocyanine iodide.

SERS has been an effective means to probe plasmonic hot-spots in AuNP oligomers. Compared with AuNPs, NPGDs not only possess large surface area but also high-density hot-spots, which are proven to provide better sensing performance. Herein, near-infrared dye 3,3′-diethylthiatricarbocyanine (DTTC) is used as a probing molecule for assessing hot-spots. The typical SERS spectrum of DTTC on NPGDs is shown in Figure 8a (black trace) with prominent Raman peaks at frequencies such as 1240, 1131, 1078, 845, 779, 627 and 500 cm^−1^. The peak at 1240 cm^−1^ is assigned to C-N-C symmetrical stretching, while the strong shoulder peak at 1078 cm^−1^ originates from C-C-C symmetrical stretching [42]. The intense Raman peaks at 1131, and 845 and 627 cm^−1^ belong to C-C stretching of various C-C bonds in the molecular skeleton. The detailed assignments based on density functional theory simulation were reported by Cai and coworkers [42].

**Figure 9:**
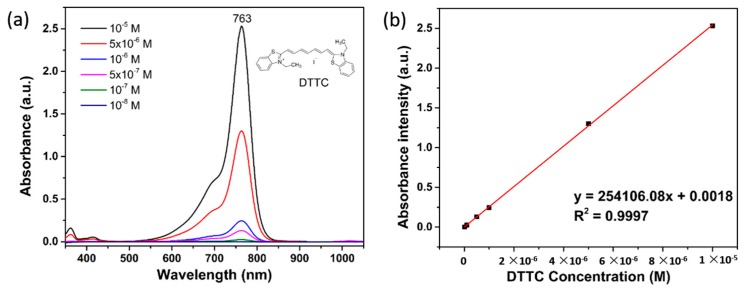
(**a**) UV-vis absorbance spectra of DTTC at different concentrations; (**b**) Linear fitting of DTTC concentration vs. absorbance intensity.

We first compare SERS spectra of DTTC adsorbed on NPGDs and 50 nm AuNPs (Figure 8a). The corresponding SEM images of self-assembled AuNPs and as-prepared NPGDs are shown in Figure 8b,c, respectively. As shown in Figure 8b, 50 nm AuNPs were assembled onto PDDA-modified Si wafer. The density of assembled AuNPs could be estimated based on the SEM image to be ~27 particles per µm^2^, while the density of NPGDs is ~9 particles per µm^2^. Since SERS spectra of both nanostructures were measured at the same conditions, the estimated Raman intensity of a single NPGD at 1131 cm^−1^ is ~60 times stronger than that of a single 50 nm AuNP. We also estimate the number of adsorbed DTTC molecules on NPGD and AuNP. First, a calibration curve was established by a linear fitting of DTTC concentrations versus absorbance intensity at 763 nm from 10^−8^ to 10^−5^ M (Figure 9). As shown in Figure 9b, the R^2^ value of the calibration curve is 0.9997 which indicates high-quality fit. NPGDs samples (~1 cm × 1 cm) were placed at the bottom of a cuvette for 1-h incubation, and the in situ absorption spectra of the solution were recorded every 20 min. The adsorption of DTTC molecules at NPGD surfaces decreases the intensity of DTTC absorbance peak. The number of adsorbed DTTC molecules is then estimated through the linear fitting equation, which is ~460,000 DTTC molecules per NPGD after incubating 1 h in 10^−5^ M solution. On the other hand, the capability of AuNP to adsorb DTTC molecules is limited by its geometrical structure, where only ~26,000 molecules per particle could be adsorbed based on calculation.

**Figure 10:**
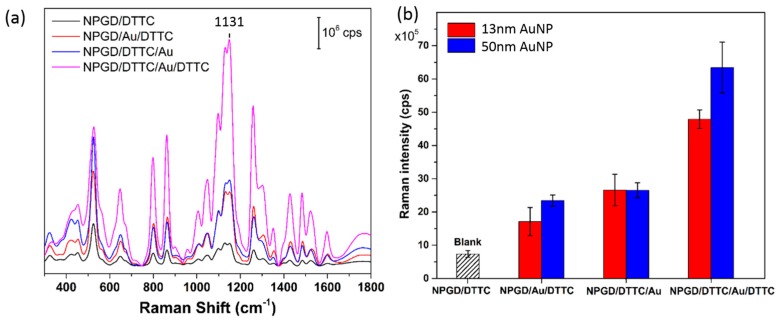
(**a**) SERS spectra of DTTC on NPGD/50 nm AuNP composites; (**b**) SERS performance comparison between 13 and 50 nm AuNPs on NPGDs.

The SERS spectra of DTTC loaded on NPGD/50 nm AuNP composites are shown in Figure 10a, and the spectra obtained from NPGD/13 nm AuNP composites are shown in Figure S2. The four spectra represent different DTTC loading strategies: NPGD loaded with DTTC as control, NPGD/50 nm AuNP composites loaded with DTTC, NPGD loaded with DTTC and then assembled with 50 nm AuNPs, and NPGD and 50 nm AuNP loaded with DTTC with the same protocols separately and then assembled together.

The 1131 cm^−1^ peak is used for quantitative evaluation. The SERS performance of 13 nm and 50 nm AuNPs on NPGDs are shown in Figure 10b. For both 13 nm and 50 nm AuNPs on NPGDs, the SERS peaks have the following relationship: NPGD/DTTC < NPGD/Au/DTTC < NPGD/DTTC/Au < NPGD/DTTC/Au/DTTC. The Raman intensity of NPGD/Au/DTTC has significantly increased compared to NPGD/DTTC. This is due to the additional hot-spots generated by assembled AuNPs. Loading DTTC before assembling AuNPs on NPGD (NPGD/DTTC/Au) resulted in higher intensity than loading DTTC after the assembly of AuNPs. The possible reason is that in the former loading strategy, more DTTC molecules are adsorbed at the gaps between the NPGD surface and the assembled AuNPs, where the hot-spots are the strongest (according to Figure 6). It is easy to understand that the last loading strategy has the strongest Raman intensity, as this method allows adsorbed DTTC molecules to occupy most of the hot-spots.

Finally, to illustrate the size effect of AuNPs in SERS performance, we now compare the Raman intensity of nanocomposites made from 13 and 50 nm AuNPs. Under the same DTTC loading strategy, NPGD/50 nm AuNP generally has better performance, mainly due to the existence of stronger hot-spots in such nanocomposites as shown in Figure 6.

## 4. Conclusions

In this paper, hybrid nanoporous gold composites have been developed for plasmonic engineering by loading AuNPs of different sizes onto NPGD array substrates. Due to coupling, the hybrid nanocomposites have red-shifted resonance peaks compared to bare NPGD array substrates. The plasmonic resonance is tunable in the near-infrared spectral range by changing the AuNP size. FDTD simulation correlates well with the experimental results, where larger AuNPs generated stronger E-field hot-spots and a stronger red-shift. The simulation also reveals that the coupling-induced hot-spots not only originate from the interactions between NPGD and AuNP, but also from between AuNPs. Hot-spots formation on various entities have been experimentally studied by surface-enhanced Raman scattering of adsorbates.

## Supplementary Materials

The following are available online at http://www.mdpi.com/1424-8220/17/7/1519/s1, Figure S1: Extinction spectra of NPGD before and after adsorbing PDDA. Figure S2: SERS spectra of DTTC on NPGD/13 nm AuNP composites.
