# Supplementary material for: Nanoporous Gold Nanocomposites as a Versatile Platform for Plasmonic Engineering and Sensing
Source: Sensors (Basel). 2017 Jun 28;17(7):1519. doi: 10.3390/s17071519 (PMC5539714; doi:10.3390/s17071519)
Supplement: Supplementary file 1 [file sensors-17-01519-s001.pdf]

Supporting Information

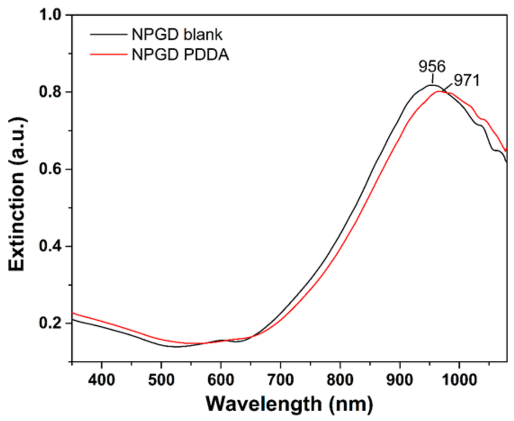

Figure S1. Extinction spectra of NPGD before and after adsorbing PDDA.

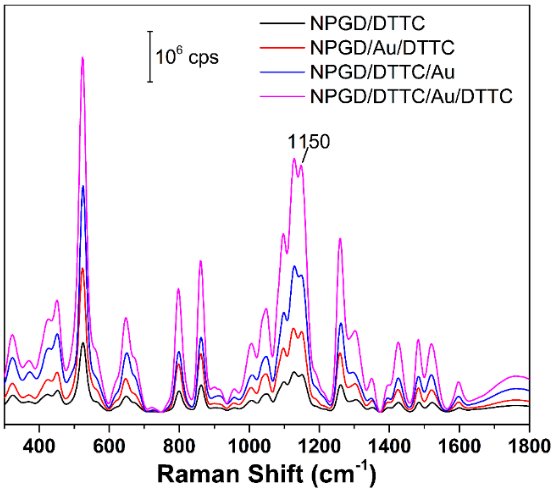

Figure S2. SERS spectra of DTTC on NPGD/13 nm AuNP composites.
